# Multimodality Cardiac Imaging in a Patient with Kawasaki Disease and Giant Aneurysms

**DOI:** 10.1155/2016/4298098

**Published:** 2016-10-31

**Authors:** Ranjini Srinivasan, Rachel Weller, Anjali Chelliah, Andrew J. Einstein

**Affiliations:** ^1^Department of Pediatrics, Division of Pediatric Cardiology, Columbia University Medical Center and Morgan Stanley Children's Hospital of New York-Presbyterian, New York, NY, USA; ^2^Department of Medicine, Division of Cardiology, Columbia University Medical Center and New York-Presbyterian Hospital, New York, NY, USA; ^3^Department of Radiology, Columbia University Medical Center and New York-Presbyterian Hospital, New York, NY, USA

## Abstract

Kawasaki disease is a well-known cause of acquired cardiac disease in the pediatric and adult population, most prevalent in Japan but also seen commonly in the United States. In the era of intravenous immunoglobulin (IVIG) treatment, the morbidity associated with this disease has decreased, but it remains a serious illness. Here we present the case of an adolescent, initially diagnosed with Kawasaki disease as an infant, that progressed to giant aneurysm formation and calcification of the coronary arteries. We review his case and the literature, focusing on the integral role of multimodality imaging in managing Kawasaki disease.

## 1. Introduction

Kawasaki disease is an acute, self-limited vasculitis that affects the small- and medium-sized arteries, with a predilection for the coronary arteries [1]. The long-term burden, followed up by the pediatric cardiologist, is coronary disease progression and possible coronary aneurysm development. Multimodal imaging can be very useful in following up these patients and looking at the progression of coronary disease. We report a case of Kawasaki disease in a patient from Japan who developed giant coronary aneurysm with thrombus and the role that imaging played in following up the progression and management of his disease. We also review the literature on Kawasaki disease including the various imaging modalities and their role in management.

## 2. Case Presentation

A 15-year-old male with a history of Kawasaki disease diagnosed as a 4-month-old infant in Japan was referred to our institution when he was 13 years old. As per his medical records, he received a dose of IVIG after approximately 2 days of symptoms with no additional doses at any point. Subsequently, he developed giant coronary artery aneurysms of the right and left coronary systems. By echocardiography at that time, the left anterior descending (LAD) measured 7.3 mm in diameter, left circumflex (LCx) 5.5 mm, and right coronary artery (RCA) 10 mm. He was treated with aspirin, dipyridamole, and ticlopidine. In July 2003, coronary angiograms showed occlusion of both the LCx and RCA with recanalization in both coronary arteries.

Later that year, after his family relocated to the United States, clopidogrel was substituted for ticlopidine and dipyridamole. In 2006, at the age of 6, after returning to Japan, repeat imaging suspected thrombus formation in the aneurysm in the LAD, and he was started on warfarin and aspirin. A scintigram at 12 years old with exercise loading showed no perfusion defects. A coronary computed tomography CT angiogram was performed in July 2013. Based on these findings, doctors in Japan recommended that he participate in low impact activities and no contact sports and with his everyday activities he reported no chest pain.

**Figure 1:**
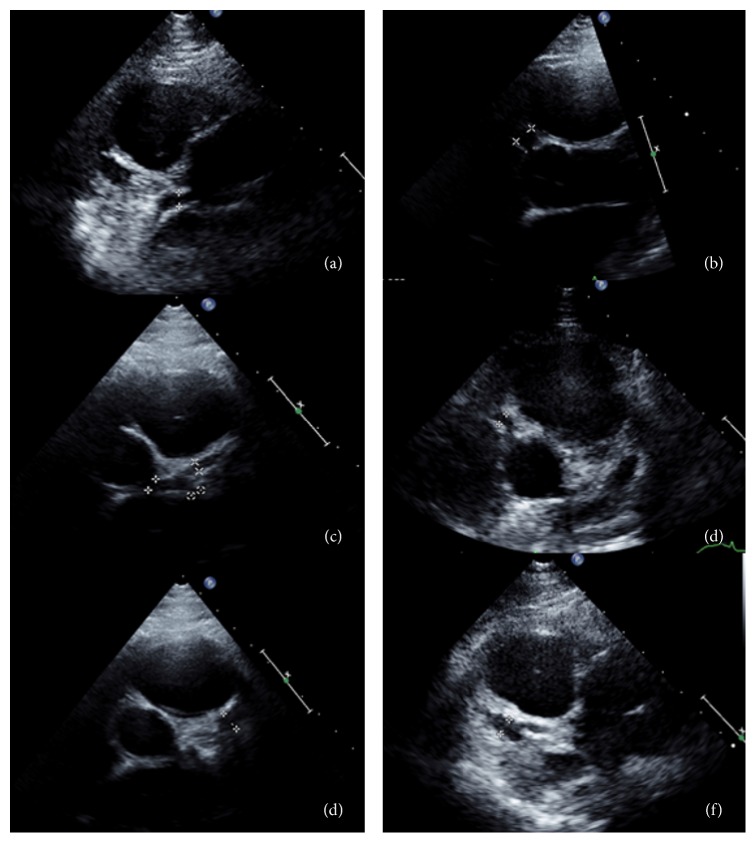
Echocardiographic images demonstrating coronary artery ectasia. Parasternal short axis views from echocardiogram. (a) Left coronary artery measured at proximal end. (b) Right coronary measured at proximal end. (c) LCA measured at distal end. (d) RCA measured at proximal end. (e) LCA at bifurcation into the left circumflex and left anterior descending coronary. (f) RCA measured at the distal end.

He was first seen in our institution at the age of 13 years, and his initial echocardiogram demonstrated a moderate-sized saccular aneurysm of the mid left anterior descending coronary artery, measuring up to 0.79 cm in diameter and 1.44 cm long. There was a small- to moderate-sized aneurysm of the right coronary artery, measuring 0.5 cm in diameter and 1.23 cm long (Figure 1). There was no evidence of thrombus, his warfarin was switched back to clopidogrel, and he remained on aspirin. Annual follow-up demonstrated that the aneurysms were stable in size, but his most recent echo showed a new echogenicity along the saccular wall of the LAD aneurysm concerning for possible thrombus.

**Figure 2:**
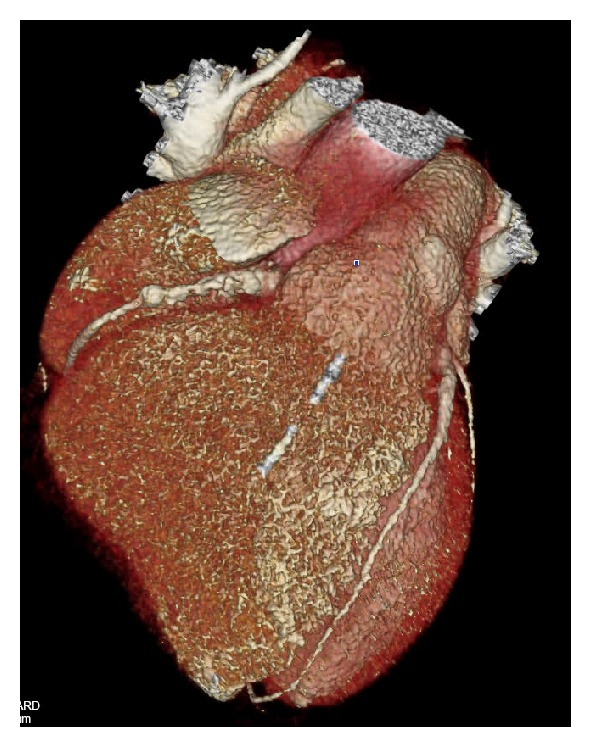
CTA Volume-Rendered Image demonstrating coronary artery aneurysms.

**Figure 3:**
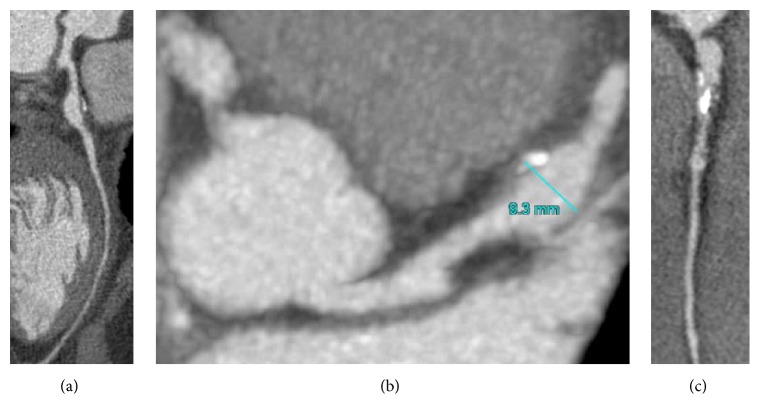
CT angiogram. (a) Curved multiplanar reformat image of the left anterior descending coronary artery. (b) Curved multiplanar reformat image of the right coronary artery. (c) Multiplanar reformat image demonstrating aneurysmal coronary arteries arising from aortic root.

Given these findings, he was referred for low-dose coronary CT angiography (Figure 2). The CT demonstrated a saccular aneurysm of the LAD with maximal diameter of 9 mm, containing mural thrombus and calcification, at the origin of the first diagonal branch (Figures 3(a) and 3(b)). There was an additional small saccular aneurysm of the LCx with diameter of 6 mm and narrowing of the RCA ostium with an associated irregular area of hypoattenuation possibly representing thrombus, followed by a long saccular calcified aneurysm extending into the mid-vessel (Figure 3(c)).

**Figure 4:**
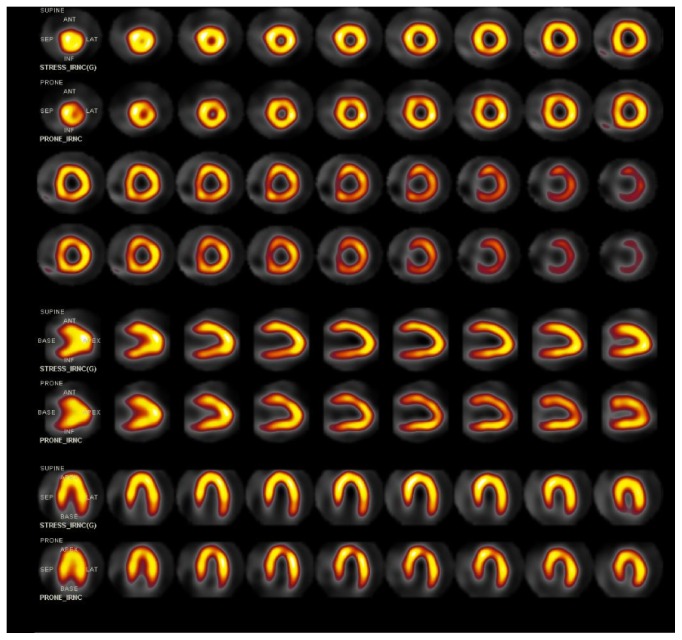
SPECT stress-only Tc-99m sestamibi myocardial perfusion imaging, demonstrating normal left ventricular perfusion. Short axis, vertical long axis, and horizontal long axis images, with images with patient supine displayed in odd rows and corresponding images with patient prone displayed in even rows.

Given the presence of coronary stenosis, he was referred for exercise treadmill stress test with SPECT myocardial perfusion imaging to evaluate the presence of inducible ischemia. His exercise capacity of 17 metabolic equivalents was excellent for age and gender. He had an appropriate hemodynamic response with no ST segment changes noted on electrocardiogram. At peak stress he was injected with 6.4 mCi of Tc99m sestamibi. Supine, prone, and gated SPECT images of the heart obtained after stress demonstrated normal myocardial perfusion with no evidence of infarction or ischemia, normal function and wall motion, and a left ventricular ejection fraction of 68% (Figure 4). We performed our patient's SPECT with a radiation dose of under 2 mSv for the entire study.

Clinically, the patient has remained asymptomatic. He is at baseline a sedentary and overweight young man, with a BMI of 23 (86th percentile). According to the Japanese Kawasaki protocol, he would be allowed moderate exercise, but he was further limited to only light intensity sports. The clopidogrel was discontinued and he was restarted on warfarin given the presence of thrombus and size of the LAD aneurysm, and he was continued on aspirin as well.

## 3. Discussion

### 3.1. Background and Epidemiology

Kawasaki disease is diagnosed based on criteria defined by the American Heart Association (AHA), with symptoms including fever for five days, with four out of five clinical features including bilateral conjunctivitis, erythematous changes of the lips and oral mucosa, changes in the extremities, rash, and cervical lymphadenopathy. In the absence of four clinical features, if 2D echocardiogram shows coronary artery disease, diagnosis can be made. Its prevalence is highest in Japan, where the annual incidence rate is 240 per 100,000 children aged up to 4 years, compared to 9 to 19 per 100,000 children in the same age range in the United States [2, 3]. Older studies from Japan in the early 1990s established mortality from Kawasaki disease of approximately 0.3% [4]. Coronary artery aneurysms, occurring in 15–25% of untreated children, are the most significant source of mortality from this disease [5]. Long-term sequelae include ischemic heart disease and, in worst case, sudden death [5].

Treatment options are primarily IVIG with high-dose aspirin as an adjunct, and studies are being done on other treatments including steroids and tumor necrosis factor-*α* inhibitors. Studies comparing the effects of high-dose IVIG (>1 g/kg), low-dose IVIG (<1 g/kg), and aspirin have shown that the incidence of coronary artery aneurysm is lowest in groups treated high-dose IVIG and aspirin, with incidence of 9% and 4% at 30 and 60 days, respectively [6]. The natural history of coronary aneurysms is regression, with about 1/2 to 2/3 of aneurysms resolving within 1-2 years after diagnosis. Saccular aneurysms have a poor prognosis compared to fusiform aneurysms [1, 5]. However, long-term studies have shown that about 3% of patients with aneurysms will develop coronary artery obstruction; the time from disease diagnosis to coronary artery stenosis varies widely from months to up to 20 years [7].

### 3.2. Infant Kawasaki Disease

The presentation in a child less than six months old differs from that in the older pediatric population. It has been reported that 28% of infants had an atypical presentation compared to 7–10% in older children, with fever and rash present but lacking other classic physical exam findings [8]. Infants bear a higher morbidity from Kawasaki disease, with 85% of these patients showing coronary artery changes [9]. It has been theorized that the vague symptoms of atypical presentation can delay diagnosis and initiation of therapy for these patients, which could explain the increased incidence of aneurysm [8]. However, it is possible that unique factors in the infantile form of disease that cause coronary artery aneurysm formation have not yet been fully elucidated. Fortunately, coronary aneurysms in infants with Kawasaki disease are more prone to aneurysm regression [5, 10].

### 3.3. Roles of Cardiac Imaging in Diagnosis and Management

The goal of imaging in Kawasaki disease is to visualize coronary artery aneurysms, calcification, and thickening of the vessels. Over time, given the risk of myocardial ischemia with these coronary artery abnormalities, one must also evaluate the evidence of scarring and remodeling of the myocardium [11, 12]. Echocardiography is generally accepted as first line imaging of these patients and is part of the diagnostic algorithm endorsed by the American Heart Association (AHA) [13]. However, its image quality and therefore diagnostic sensitivity are highly operator-dependent. Although younger children typically have good acoustic windows, it may be difficult to adequately visualize the coronary arteries in older children [13]. Regardless, the reported sensitivity and specificity for identifying coronary aneurysms by echocardiography are high, 95 and 99%, respectively, in one recent study [14]. For identifying stenosis, sensitivity and specificity were in the ranges 80–85% and 97-98%, respectively [14]. Dobutamine stress echocardiography is another modality often used to look for evidence of wall motion abnormality secondary to coronary stenosis. However, wall motion abnormalities by stress echo are only detected in about 50% of patients with more than 50% coronary occlusion and therefore are a late indicator of disease [13].

One obvious limitation of echocardiography is its inability to visualize the coronary artery system distal to its largest vessels. Invasive coronary angiography is an important modality in assessing the coronary arteries and evaluating stenosis, aneurysm, collateral formation, or thrombus with higher diagnostic accuracy than echocardiography [15]. CT and magnetic resonance imaging (MRI) have further changed the dynamic of noninvasive imaging by providing visualization of the entire coronary artery system.

Using CT and MRI to image the coronaries presents its own set of challenges. Both modalities require that the motion associated with cardiac contraction and respiration be minimized to produce optimal images. End-diastole is commonly the optimal time for acquiring an image due to minimization of motion [16]. In both CT and MRI, breath holding may be used to decrease respiratory motion, although free breathing techniques are becoming more common when using a real-time navigator for respiratory motion tracking and gating [16]. CTA and MRI can clearly show coronary artery aneurysm, the distal coronary arteries, as well as calcifications and thrombus within the coronary artery and have been proven superior to echocardiography in this aspect [13]. Multislice CT has high sensitivity and specificity for detection of coronary artery stenosis [13]. When specifically looking for evidence of coronary luminal stenosis, CT angiography provides slightly better imaging than coronary magnetic resonance angiography (MRA) [11]. A prospective multicenter trial in adults established that coronary CTA is highly accurate for coronary artery stenosis at thresholds of 50% and 70% stenosis and has a 99% negative predictive value for coronary stenosis [17]. However, the radiation required in young pediatric patients and the secondary risks from radiation exposure made many practitioners reluctant to screen frequently using this modality. Nevertheless, with optimal technique it is possible to perform CT angiography with a radiation effective dose of less than 1 mSv, equivalent to several months' background exposure to environmental radiation.

MRA has also become a popular imaging option and eliminates the concerns of ionizing radiation exposure that are common with CTA. MRI allows visualization of the coronary anatomy and also provides a quantitative assessment of cardiac function [18]. Cardiac MRI can also evaluate myocardial fibrosis using techniques such as late gadolinium enhancement and, more recently, T1 and T2 mapping [12]. Compared to the traditional use of coronary angiography, one recent study showed that the measurements of coronary artery ectasia were statistically similar between angiography and MRA [19]. The subclinical myocardial inflammation is missed by other imaging modalities such as CT or SPECT [18]. The potential need for anesthesia in young children is a limitation. A disadvantage of cardiac MRI is that the spatial resolution tends to be lower than CTA, and in particular visualization of the distal coronary and branch arteries is often challenging [11].

The use of single-photon emission computed tomography (SPECT) in this patient population allows one to evaluate myocardial perfusion at exercise stress and at rest, assess ischemia in patients with coronary stenosis, and identify scarring in patients with thrombosed coronary arteries. SPECT as traditionally performed utilizes relatively high doses of ionizing radiation, raising similar concerns to CT, and is therefore avoided in the pediatric population. However, SPECT too can be performed using low-dose technique to limit the effective dose of radiation to under 2 mSv each for stress and, if needed, rest imaging.

### 3.4. Management and Intervention

Primary management of Kawasaki disease is directed toward resolving fever and inflammation and preventing coronary disease. In the acute phase, high-dose aspirin is used as both anti-inflammatory and antiplatelet therapy, and IVIG adds a synergistic anti-inflammatory effect [5]. Previous studies have shown that combined treatment with aspirin and IVIG markedly reduces the incidence of coronary artery aneurysm; one large meta-analysis found coronary artery aneurysms at 60 days in only 2.4% of patients who had received a single dose of greater than 1 g/kg of IVIG and aspirin as initial therapy. There was no statistically significant difference in aneurysm development rates between patients on long-term high-dose versus low-dose aspirin [6].

Once coronary artery involvement occurs and thrombus is noted by imaging, treatment is directed towards antiplatelet therapies such as clopidogrel and aspirin. Clopidogrel, a platelet glycoprotein receptor inhibitor, has shown promise in preventing platelet aggregation and has been used in conjunction with TPA to restore flow to occluded vessels [5]. TPA, streptokinase, and urokinase are not commonly used, however, and with its discussion in the literature limited to case reports. In rapidly expanding aneurysms, particularly in the acute phase, heparin or warfarin is often utilized, especially once thrombus has been identified [10]. The incidence of obstruction secondary to giant coronary aneurysm has been studied extensively. In the era prior to IVIG treatment when these complications were more prevalent, one institutional study identified coronary artery aneurysm obstruction in 30% of patients within 4 years of disease onset. This study noted that antiplatelet therapy did decrease the incidence of obstruction in patients compared with other studies with similar numbers of patients [20].

Interventional and surgical techniques have been explored for management of coronary artery obstruction, particular in Japan where the disease prevalence is more common. Long-term studies in the 1970s and 1980s emphasized the role of invasive coronary angiography in following up the progression of coronary artery aneurysm and recommended CAG over periodic intervals to monitor for late sequelae [1]. However, with the advent of less invasive imaging modalities, such as cardiac CMR or CTA, invasive angiography became less preferred for routine surveillance due to the potential for catheter related complications [5, 16]. There are no randomized controlled studies of intervention for Kawasaki-associated coronary artery obstruction in children; therefore the pediatric literature derives guidelines from the adult studies on acute coronary syndromes [5]. Overall, guidelines for surgical intervention are not well defined, especially since coronary disease in children with Kawasaki disease tends to regress over time compared to adults [21]. The indication for CABG is typically based on clinical presentation including symptoms related to myocardial infarction and ischemia, and by imaging findings of ischemia and decreased ventricular function [7]. An occlusive lesion in one or more of the coronary arteries causing ischemia is an indication for intervention [22]. Resection of an aneurysm is difficult because of technical issues with reconstructing branch vessels, and even partial resection of an aneurysm may produce high rates of occlusion in the future [5, 22]. Aneurysmal excision is associated with higher mortality compared to CABG [22].

Cardiac transplant may be the only remaining option in settings where there is severe myocardial dysfunction, refractory severe ventricular arrhythmia, or severe distal multivessel occlusive coronary artery disease [23].

## 4. Conclusion

This patient initially presented as an infant and received very early treatment with IVIG and aspirin, and despite the lack of symptom recurrence he developed saccular aneurysms. Furthermore, although he received long-term treatment with clopidogrel and aspirin, he has developed thrombus within his aneurysms.

The multiple imaging modes in this case contributed in complementary ways to furthering our understanding of the progression of this patient's disease. Based on the AHA and Japanese published risk stratification for patients with Kawasaki disease, the group consensus at our institution was to pursue expectant management, following him up every six months by echocardiography and annually with a low-radiation nuclear stress test [5]. A new finding of myocardial ischemia or significant progression of aneurysms could prompt percutaneous or surgical intervention [5]. The role of imaging would be crucial in identifying evidence of ischemia or increasing size of aneurysms. In addition, based on AHA and Japanese criteria, the patient is moderately exercise restricted and is not allowed to play competitive or contact sports since he is currently on antiplatelet therapy and warfarin. This case illustrates the role that imaging has played in the evolving management of Kawasaki disease and the future of this disease that continues to be a challenge for pediatric cardiologists, in both diagnosis and management.
